# Association study of genetic variants at TTC32‐WDR35 gene cluster with coronary artery disease in Chinese Han population

**DOI:** 10.1002/jcla.23594

**Published:** 2020-10-02

**Authors:** Ying Xu, Yang Zhuo, Mengliang Ye, Mengmeng Li, Xiaojun Tang, Li Zhou

**Affiliations:** ^1^ Department of Epidemiology School of Public Health and Management Chongqing Medical University Chongqing China; ^2^ Department of Health Statistics School of Public Health and Management Chongqing Medical University Chongqing China

**Keywords:** coronary artery disease, expression quantitative trait locus, severity, single nucleotide polymorphism

## Abstract

**Background:**

TTC32‐WDR35 gene cluster has been genome‐wide significantly associated with coronary artery disease (CAD). However, the common variants in this region contributing to CAD risk remain elusive.

**Methods:**

We performed a case‐control study enrolling 935 CAD cases and 935 age‐sex‐frequency‐matched controls from unrelated southwest Chinese Han population. Five variants were determined by TaqMan assay.

**Results:**

This study indicated that rs721932 CG genotype was associated with CAD risk (OR = 0.68, 95% CI: 0.54‐0.86; *P* = .001). Stratified analysis showed that the risk associated with rs12617744 AA genotype was robust in male (OR = 0.62, 95% CI: 0.42‐0.93, *P* = .02). The gene dosage of the risk allele at rs12617744 showed a significant association with left circumflex artery disease (*P* = .027) and the number of vascular lesions in patients (*P* = .034). Moreover, the gene dosage of rs721932 risk allele was associated with vascular lesion numbers (*P* = .048) and the progression of CAD (*P* = .028). Compared with carriers of major alleles, the AA genotype of rs12617744 and GG genotype of rs721932 were both associated with plasma HDL level (*P* = .009 and 0.004, respectively). Expression quantitative trait locus (eQTL) results showed significantly different TTC32 expression of subjects as a function of SNPs (rs2278528, rs7594214, and rs721932) genotype in the artery. Besides, FPRP analysis did support the strong links between polymorphisms and CAD risk.

**Conclusions:**

SNP rs721932 at TTC32‐WDR35 Gene Cluster was associated with CAD risk, and rs12617744 was associated with the risk of CAD among males. Both SNPs may contribute to the regulation of plasma HDL levels and possibly to the severity of CAD in Chinese Han population.

## Graphical abstract

SNPs at TTC32‐WDR35 Gene Cluster are significantly associated with CAD risk. Both SNPs may contribute to regulation of plasma HDL levels and possibly to the severity of CAD in a Chinese Han population.

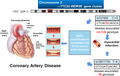

**Abbreviations**
BMI: body mass index
CAD: coronary artery disease
CI: confidence interval
GWASs: genome‐wide association studies
HDL: high‐density lipoprotein
Het: heterozygote
Hom Maj: homozygote of majority
Hom Min: homozygote of minority
LAD: left anterior descending artery disease
LCX: left circumflex artery disease
LD: linkage disequilibrium
LDL: low‐density lipoprotein
LM: left main coronary artery disease
OR: indicates odds ratio
RAD: right coronary artery disease
SNP: single nucleotide polymorphisms
TC: total cholesterol
TG: triglyceride
TRP: tetratricopeptide repeat motif
WHO: World Health Organization

## 1 INTRODUCTION

Coronary artery disease (CAD) is the predominant cause of mortality and disability worldwide.^1^, ^2^, ^3^ The incidence of CAD has increased considerably in China over the past few years. To date, genome‐wide association studies (GWASs) have highlighted at least 161 loci associated with CAD risk at genome‐wide significance.^4^, ^5^, ^6^, ^7^, ^8^ However, most of the GWASs have centered on individuals of European ancestry. Therefore, more studies in the Chinese population are urgently needed to improve the understanding of the genetic architecture of CAD among different populations. The largest GWAS of CAD in Asia so far identified a single nucleotide polymorphism (SNP) at 2p24.1 was associated with the susceptibility to CAD in Chinese.^5^ Meanwhile, research also reported that another variant at 2p24.1 was significantly associated with CAD in Europeans.^8^ Both studies highlight that the chromosome 2p24.1 region is a susceptible locus to CAD. Therefore, we fine mapped this region through UCSC Genome Brower (http://genome.ucsc.edu/) and showed great interest in the TTC32‐WDR35 gene cluster, which is the protein‐coding genes rich region nearest to the reported CAD‐associated SNPs.

TTC32 encodes the TRP‐containing protein (tetratricopeptide repeat motif, TRP) whose TRP motif mediates binding to other peptides.^9^ WDR35 encodes a protein, the member of the WD‐repeat protein family^10^ involving in cell cycle progression, signal transduction, apoptosis, and gene regulation.^11^, ^12^ Studies confirmed that the expression of WDR35 was related to body weight, FBG, and glycosylated haemoglobin^13^ and that nuclear factor‐kappa B signaling pathway was involved in the expression of WDR35.^13^ Both genes are involved in a variety of cellular processes. Additionally, in this region are extensively transcripted several long non‐coding RNAs (lncRNAs), which are a class of non‐coding RNAs 200 nt or longer in length. Accumulating studies have reported that lncRNAs were potentially implicated in the regulation of diverse atherosclerosis events involving the modulation of protein‐coding genes in proximity.^14^, ^15^ However, the relationship of the TTC32‐WDR35 gene cluster and CAD risk was uncertain, and there was a lack of evidence concerning the effect of SNPs in the TTC32‐WDR35 gene cluster on cardiovascular risk.

To further explore the associations between polymorphism in the locus and CAD risk and to facilitate the determination of genuinely associated variants and the establishment of complete molecular pathways underlying the pathogenesis of CAD, we conducted a CAD case‐control study in Han Chinese to test five SNPs (rs12617744, rs2278528, rs7594214, and rs3731661, rs721932) in TTC32‐WDR35 gene cluster.

## 2 METHODS

### 2.1 Study population

The study population was composed of 935 case‐patients and 935 age‐ and sex‐frequency‐matched controls. All enrolled subjects were unrelated ethnic Han Chinese derived from Chongqing City (in Southwest China). Inclusion criteria for CAD populations were either: (a) presence of stenosis ≥50% in at least one major segment of coronary arteries (the right coronary artery, left circumflex, or left anterior descending arteries) by coronary angiography; (b) typical symptoms of angina pectoris, electrocardiographic changes and elevations of cardiac enzymes according to World Health Organization criteria^16^; (c) a documented history of coronary artery bypass graft or percutaneous coronary intervention. Patients with congenital heart disease, cardiomyopathy, and valvular disease were excluded. The controls, residing in the same communities as the cases, were determined to be free of CAD, cardiovascular and cerebrovascular disease, and peripheral atherosclerotic arterial disease by medical history, clinical examinations, and electrocardiography. Subjects with severe liver and/or kidney disease were excluded. Structured questionnaires were accomplished by trained interviewers to collect information on demographic variables, medical history, medications use, and lifestyle factors.

The subjects were defined as smokers and nonsmokers. Those who had smoked fewer than 100 cigarettes in the past lifetime were classified as nonsmokers; otherwise, they were identified as smokers. Alcohol consumption was classified into two categories: drinkers and nondrinkers. Subjects that reported drinking any alcoholic beverage “less than once a year” or “never” were classified as nondrinkers; otherwise, they were coded as drinkers. Body mass index (BMI) was calculated as weight in kilograms divided by the square of height meters.

### 2.2 Angiography

The coronary angiograms were evaluated by two independent, experienced angiographers who were both unaware of the genotype results according to the Judkins technique through the right radial artery. The vessels were scored as follows: stenosis <50% in any major coronary artery was classified as 0‐vessel disease; a significant coronary lesion was defined as showing ≥50% stenosis in the luminal diameter in one or more coronary arteries. The major coronary artery disease, involving left main coronary artery disease, left anterior descending artery disease, right coronary artery disease, and left circumflex artery disease, was described in a previous study.^17^

### 2.3 SNP selection

A total of five SNPs within the 2p24.1 region, including rs12617744, rs2278528, rs7594214, rs3731661, and rs721932, were selected in this study by Haploview 4.2. Other common variants in this region can be well captured by this set of five SNPs in this region (*r* ^2^ > .8). The selected SNPs met the following inclusion criteria: minor allele frequency (MAF) ≥0.1 in Chinese Han population of the HapMap project (the Phase II database). To better capture insight into the linkage disequilibrium (LD) pattern, the LD of the five SNPs is summarized in Figure S1.

### 2.4 Genotyping

Fasting venous blood was collected from each participant in 5‐ml EDTA tubes, and the genomic DNA was isolated with a Tiangen DP319‐02 kit (Tiangen Company, Beijing, China). Genotyping was performed with the TaqMan Genotyping Assay Systems and ABI PRISM 7900HT (Applied Biosystems, Foster City, CA, USA), in 384‐well format. The TaqMan Assay kit was purchased from Applied Biosystems (Foster City, CA, USA). It included the forward target‐specific polymerase chain reaction (PCR) primer, the reverse primer, and the TaqMan MGB probes labeled with two special dyes: FAM and VIC. PCRs were carried out in reaction volume of 5 μL containing 5 ng DNA, 2.5 μL 2 × Taqman universal PCR Master MixNo AmpErase UNG (Applied Biosystems, Foster City, CA, USA), 0.125 μL 40 × Assay MIX. PCR conditions included 95°C for 10 minutes, followed by 40 cycles of 15 seconds at 92°C and 1 minutes at 60°C. Two blank controls (DNA hydration solution) and two replication quality control samples were included in each run, and two replicate samples were genotyped with 100% concordance. A successful genotyping rate of more than 95% was achieved for the tested SNPs. Genotypes were automatically detected by analysis software version 2.2.1 (SDS 2.2.1).

### 2.5 Statistical analysis

Continuous variables were expressed as the mean value ± SD. Categorical variables were reported as frequencies with percentages. Normal distribution of data was determined using the Kolmogorov‐Smirnov Normality test. Data with a normal distribution were analyzed by Student's *t* test, and those with unequal variance or significantly deviated from normal distribution were compared by a Mann‐Whitney rank sum test. Categorical values were analyzed by the chi‐square test, which was also used to check for deviation of genotype distribution from Hardy‐Weinberg equilibrium. The associations between variants in the TTC32‐WDR35 gene locus and CHD risk were evaluated by computing the odds ratios (ORs) and 95% confidence intervals (CIs) using the binary logistic regression with adjustment for age, sex, smoking, drinking, blood pressure, and diabetes. The relationship between SNP genotype and the location of CHD lesions were analyzed by Kendall rank correlation analysis. The association between the numbers of affected vessels in CAD patients and genotypes of the risk variants was assessed with chi‐squared test. The lipid levels were log‐transformed before analysis. Multiple linear regression with adjustment for age, gender, smoking, and drinking was performed to assess the associations between the plasma lipid levels and SNP genotypes. Haploview version 4.2 was used to estimate linkage disequilibrium with data derived from 1000 Genomes datasets.^18^ The probability level accepted for significance was *P* < .05. All data analyses were carried out with the statistical analyses software package SPSS 22.0 (SPSS Inc, Chicago, IL, USA).

Furthermore, as the relevant information of previous studies,^19^, ^20^, ^21^ the false‐positive report probability (FPRP) test was adopt to assess the credibility of statistically significant associations of the true genetic relationship probability. We set 0.2 as an FPRP threshold, power OR with 0.67/1.50 (protective/risk effects), and prior probability levels with “0.25, 0.1, 0.01, 0.001, 0.0001.” Only the significant result with an FPRP value <0.2 under the prior probability level of 0.1. was considered a noteworthy finding.

## 3 RESULTS

### 3.1 Characteristics of the subjects and variants

The general characteristics of the study samples are demonstrated in Table 1. The age and sex were frequency matched between the cases and controls. The proportion of smokers was significantly higher in cases than in controls (25.3% vs 15.6%). The percentage of drinkers was significantly lower in cases than in controls (11.1% vs 17.4%). There was no significant difference in BMI and total cholesterol (TG) level between the two groups. The lower total cholesterol (TC), high‐density lipoprotein (HDL), low‐density lipoprotein cholesterol, and higher fasting glucose were observed in cases than in controls.

**TABLE 1:** General characteristic of coronary artery disease (CAD) cases and controls

Variables	Cases (N = 935)	Controls (N = 935)	*P* value
Sex, m/f, (%)	48.5/51.5	50.4/49.6	.41
Age, mean ± SD	66.4 ± 10.7	65.5 ± 12.8	.13
BMI, kg/m^2^	23.4 ± 3.0	23.6 ± 3.2	.52
Smoking, no/yes, %	74.7/25.3	84.4/15.6	<.01
drinking, no/yes,%	88.9/11.1	82.6/17.4	<.01
Fasting glucose, mmol/L	6.7 ± 2.8	6.1 ± 2.5	<.01
TC, mmol/L	4.3 ± 1.2	4.8 ± 1.1	<.01
TG, mmol/L	1.6 ± 1.2	1.5 ± 1.1	.34
HDL, mmol/L	1.2 ± 0.4	1.3 ± 0.5	.02
LDL mmol/L	2.5 ± 1.0	2.9 ± 0.9	<.01
Coronary angiography	388	0	…
0‐vessel disease, %	143	…	…
1‐vessel disease, %	121	…	…
2‐vessel disease, %	68	…	…
3‐vessel disease, %	56	…	…
LM, %	20	…	…
LAD, %	225	…	…
RAD, %	116	…	…
LCX, %	109	…	…

Abbreviations: BMI, body mass index; CAD, indicates coronary artery disease; HDL, high‐density lipoprotein; LAD, left anterior descending artery disease; TG, triglyceride; LCX, left circumflex artery disease; LDL, low‐density lipoprotein; LM, left main coronary artery disease; RAD, right coronary artery disease; TC, total cholesterol.

The proportion of participants reported taking lipid‐regulating medications such as statins and fibrates in the whole cases and controls were 28.9% and 1.2%, respectively. The angiographic data presented the number and location of the diseased vessels in 388 coronary angiographic patients.

The baseline characteristics of the five selected variants in the Chinese Han population are presented in Table S2. Notably, two SNPs were located in the chromosome region of lncRNAs transcription. The SNP rs2278528 was located in the exon of lncRNA AC013400.1, rs721932 in the intron of lncRNA AC079145.1. The minor allele frequencies (MAF) of all the 5 SNPs were similar to those from the HapMap Project Chinese Han data.

### 3.2 Association between the variants and the risk of CAD

The allelic and genotypic distribution of the SNPs rs12617744, rs2278528, rs7594214, rs3731661, and rs721932 between CAD patients and controls are summarized in Table 2. As shown in Table 2, the univariate analysis indicated that genotype CG of SNP rs721932 was significantly associated with the risk of CAD (OR = 0.77, 95% CI: 0.64‐0.93, *P* = .01). After adjustment for conventional CAD risk factors such as age, gender, smoking, drinking, blood pressure, and diabetes, the CG genotype of rs721932 still presented a significant association with CAD risk (OR = 0.68, 95% CI: 0.54‐0.86; *P* = .001). A significant association (*P* = .0016) in rs721932 genotype was observed in the genetic dominant model. When we examined MI separately, the association with this SNP was still significant (OR of MI for the CG genotype = 0.53, 95% CI: 0.36‐0.78, *P* = .001) (Table S3). For the SNP rs12617744, the univariate OR of MI for the TA genotype was 1.49 (95% CI: 1.03‐2.15, *P* = .04) (Table S3). However, the association between rs12617744 and MI disappeared under multivariate analysis.

**TABLE 2:** Association of SNPs in TTC32‐WDR35 gene cluster with risk of CAD in a Chinese Han population

	Cases N (%)	Controls N (%)	HWE	Crude OR(95% CI)	*P*	Adjusted OR^a^ (95% CI)	Adjusted *P* ^a^
rs12617744							
TT	323 (35.4)	314 (35.7)	0.96	1		1	
TA	450 (49.3)	414 (47.1)		1.06 (0.86‐1.30)	.60	0.96 (0.75‐1.23)	.76
AA	139 (15.2)	151 (17.2)		0.90 (0.68‐1.18)	.43	0.98 (0.69‐1.38)	.89
Dominant	589 (64.6)	565 (64.3)		1.01 (0.84‐1.23)	.89	0.97 (0.77‐1.23)	.81
Recessive	773 (84.8)	728 (82.8)		1.15 (0.90‐1.48)	.27	1.00 (0.74‐1.37)	.99
rs2278528							
CC	340 (37.2)	319 (35.0)	0.14	1		1	
CG	442 (48.3)	460 (50.4)		0.90 (0.74‐1.10)	.31	0.87 (0.68‐1.11)	.26
GG	133 (14.5)	133 (14.6)		0.94 (0.71‐1.25)	.66	1.01 (0.72‐1.42)	.96
Dominant	575 (62.8)	593 (65.0)		0.91 (0.75‐1.10)	.33	0.90 (0.72‐1.14)	.38
Recessive	782 (85.5)	779 (85.4)		1.00 (0.77‐1.30)	.98	0.99 (0.98‐1.00)	.17
rs7594214							
TT	653 (70.7)	635 (69.2)	0.73	1		1	
TC	243 (26.3)	263 (28.7)		0.90 (0.73‐1.10)	.31	0.90 (0.70‐1.15)	.40
CC	27(2.9)	19 (2.0)		1.38 (0.76‐2.51)	.29	2.24 (1.00‐5.01)	.05
Dominant	270 (29.2)	282 (30.8)		0.93 (0.76‐1.14)	.48	0.96 (0.75‐1.22)	.73
Recessive	896 (97.1)	898 (97.9)		0.70 (0.39‐1.27)	.24	0.99 (0.98‐1.00)	.12
rs3731661							
GG	506(54.6)	526 (57.5)	0.32	1		1	
GA	363 (39.2)	341 (37.3)		1.11 (0.91‐1.34)	.30	1.11 (0.88‐1.40)	.39
AA	57(6.2)	48(5.2)		1.23 (0.83‐1.85)	.31	0.98 (0.60‐1.61)	.93
Dominant	420 (45.4)	389 (42.5)		1.12 (0.93‐1.35)	.22	1.09 (0.87‐1.37)	.43
Recessive	869 (93.8)	867 (94.8)		0.84 (0.27‐1.25)	.40	0.99 (0.98‐1.00)	.16
rs721932							
CC	545 (58.7)	486 (52.9)	0.001	1		1	
CG	339 (36.5)	393 (42.8)		0.77 (0.64‐0.93)	.01	0.68 (0.54‐0.86)	.001
GG	44 (4.7)	39 (4.2)		1.01 (0.64‐1.58)	.98	1.05 (0.62‐1.79)	.86
Dominant	383 (41.3)	432 (47.1)		0.79 (0.66‐0.95)	.012	0.70 (0.56‐0.87)	.0016
Recessive	884 (95.3)	879 (95.8)		0.89 (0.57‐1.39)	.61	0.81 (0.48‐1.36)	.42

Abbreviations: OR indicates odds ratio; CI, confidence interval; HWE, Hardy‐Weinberg equilibrium.

^a^ Data were calculated by logistic regression analysis with adjustment for age, sex, smoking, drinking, blood pressure and diabetes mellitus.

Stratified analysis indicated a significant association between SNPs (rs12617744 and rs721932) and risk of CAD (Table 3). Our results showed that genotype AA of the SNP rs12617744 was associated with risk of CAD among males and smokers (OR = 0.62, 95% CI: 0.42‐0.93, *P* = .02 and OR = 0.45, 95% CI: 0.23‐0.88, 0.02, respectively). We combined the subjects carrying G allele of rs721932 in stratification analysis due to the small number of subjects with GG genotype. The results indicated that genotypes CG and GG carriers of rs721932 had significant association with CAD risk in males (OR = 0.68, 95% CI: 0.52‐0.89, *P* = .004), older than 60‐year‐old subjects (OR = 0.79, 95% CI: 0.63‐0.99, *P* = .04), and drinkers (OR = 0.53, 95% CI: 0.31‐0.89, *P* = .02). After adjusting for conventional CAD risk factors such as age, gender, smoking, drinking, blood pressure, and diabetes mellitus, the AA genotype of rs12617744 still had significant associations with CAD in males (OR = 0.59, 95% CI: 0.36‐0.97, *P* = .04) (Table S4). Furthermore, when multiplicative interaction was tested for each possible pair of the SNP, we found significant interactions between rs12617744 and gender (*P* = .01) (Table S4).

**TABLE 3:** Stratification analysis for association between two SNPs genotypes and risk of CAD

	rs12617744 OR (95% CI), *P* value			rs721932 OR (95% CI)*, *P* value	
	TT	AT	AA	CC	CG + GG
Gender					
Male	1.00	0.97 (0.72‐1.30)	0.62 (0.42‐0.93)	1.00	0.68 (0.52‐0.89)
		0.83	0.02		0.004
Female	1.00	1.12 (0.84‐1.51)	1.29 (0.87‐1.91)	1.00	0.90 (0.70‐1.17)
		0.44	0.21		0.45
Age, y					
≤60	1.00	1.27 (0.88‐1.84)	0.91 (0.56‐1.51)	1.00	0.79 (0.57‐1.11)
		0.20	0.73		0.17
>60	1.00	0.94 (0.73‐1.21)	0.89 (0.63‐1.25)	1.00	0.79 (0.63‐0.99)
		0.64	0.48		0.04
Smoke status					
Yes	1.00	0.82 (0.51‐1.32)	0.45 (0.23‐0.88)	1.00	0.76 (0.49‐1.17)
		0.41	0.02		0.21
No	1.00	1.03 (0.81‐1.31)	1.03 (0.75‐1.42)	1.00	0.82 (0.66‐1.01)
		0.80	0.84		0.06
Drink status					
Yes	1.00	0.76 (0.43‐1.34)	0.58 (0.26‐1.30)	1.00	0.53 (0.31‐0.89)
		0.35	0.19		0.02
No	1.00	1.01 (0.81‐1.27)	0.91 (0.67‐1.24)	1.00	0.85 (0.70‐1.05)
		0.92	0.57		0.13

### 3.3 Association between two CAD‐related variants and the severity of CAD

**FIGURE 1:**
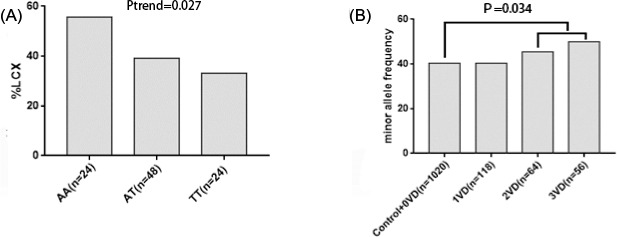
Analysis of rs12617744 and coronary artery disease (CAD) severity. A, The proportion of CAD patients with left circumflex artery disease (LCX) as a function of genotype. B, Association between the frequencies of the rs12617744 risk allele and number of vascular lesions. LCX indicates left circumflex artery disease; 0VD, 0‐vessel disease; 1VD, 1‐vessel disease; 2VD, 2‐vessel disease; and 3VD, 3‐vessel disease

**FIGURE 2:**
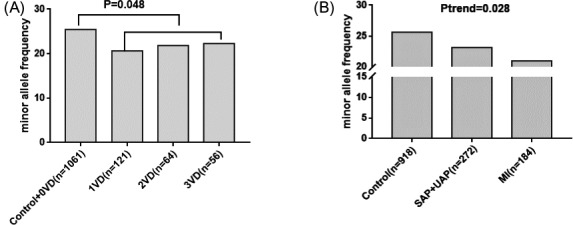
Analysis of rs721932 and coronary artery disease (CAD) severity. A, Association between the frequencies of the rs721932 minor allele and number of vascular lesions; B, Association between the frequencies of the rs721932 minor allele and the progress of CAD. SAP indicates stable angina pectoris; UAP, unstable angina pectoris; MI, myocardial infarction

There was a strong association between the proportions of CAD cases with left circumflex artery disease and increasing gene dosage of rs12617744. Along with an increasing allele A frequency of rs12617744, the proportion of CAD patients with left circumflex artery disease was significantly and gradually higher (*P* = .027; Figure 1A). We explored whether the gene dosage of rs12617744 and rs721932 was associated with vascular lesions. The results indicated that the higher gene dosage carriers of the rs12617744 A allele had a significantly greater chance of having more than two vessel coronary lesions compared with the control and 0‐vessel disease subjects (OR = 1.33, 95% CI: 1.02‐1.74; *P* = .034; Figure 1B). The results also showed that the lower gene dosage carriers of the rs721932 G allele had significantly smaller odds of developing vessel coronary lesions compared with the control and 0‐vessel disease groups (OR = 0.78, 95% CI: 0.60‐1.00, *P* = .048; Figure 2A). We further assessed the effect of SNPs on the development of CAD. The result showed that there was a significantly decreasing trend in rs721932 minor allele frequency among control, angina pectoris, including SAP and UAP, and MI groups (*P* = .028, Figure 2B).

### 3.4 Association between the SNPs and plasma lipid levels

Two CAD‐associated SNPs (rs12617744 and rs721932) were also tested for associations with plasma TG, HDL, TC, LDL levels only in the control subjects to minimize the effects of lipid‐lowering drugs. As shown in Table 4, both the SNPs were significantly associated with the HDL levels (*P* = .009 for rs12617744 and 0.004 for rs721932, respectively).

**TABLE 4:** Associations between SNPs rs12617744 and rs721932 in TTC32‐WDR35 gene cluster and plasma lipid level in Chinese

Genotypes	^a^mg/dL			*P*
	Hom Min	Het	Hom Maj	
rs12617744(T > A)				
TG	1.27 (1.02‐1.87)	1.22 (0.89‐1.8)	1.24 (0.92‐1.78)	.093
HDL	1.07 (0.82‐1.44)	1.24 (0.95‐1.63)	1.23 (0.90‐1.56)	.009
TC	4.71 (4.11‐5.43)	4.79 (4.15‐5.43)	4.84 (4.28‐5.42)	.667
LDL	2.88 (2.30‐3.41）	2.85 (2.29‐3.43）	2.86 (2.32‐3.29）	.569
rs721932 (C > G)				
TG	1.14 (0.96‐1.54)	1.26 (0.93‐1.78)	1.24 (0.92‐1.80)	.103
HDL	1.14 (0.73‐1.37)	1.22 (0.92‐1.62)	1.24 (0.92‐1.59)	.004
TC	4.45 (3.75‐4.93)	4.78 (4.11‐5.29)	4.82 (4.20‐5.52)	.403
LDL	2.56 (1.87‐3.17）	2.83 (2.28‐3.33)	2.87 (2.35‐3.37)	.605

Note

Hom Maj indicates homozygote of majority; Het, heterozygote; Hom Min, homozygote of minority.

^a^ Data are presented as median (interquartile range) of subjects.

### 3.5 Association between SNPs and TTC32 expression levels

**FIGURE 3:**
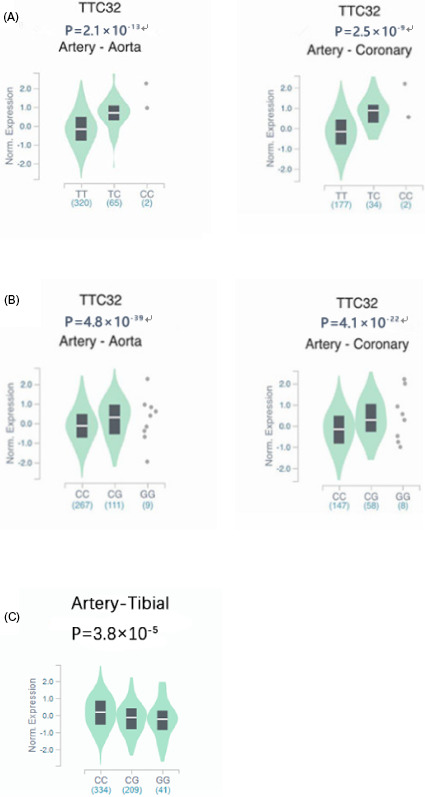
Expression Quantitative Trait Loci (eQTL) analysis using data in Genotype‐Tissue Expression Project (GTEx) database. A, the TTC32 gene expression level as a function of **rs2278528** genotype in coronary artery and aorta artert; B, the TTC32 gene expression level as a function of **rs7594214** genotype in coronary arteryand aorta artert; C, the gene expression level of TTC32 as a function of **rs721932** genotype in tibial artery

Expression quantitative trait locus (eQTL) analyses have been utilized to identify SNPs that conduce to gene expression levels, thereby providing clues to putative causative genes and reasonable biological mechanism. In the Genotype‐Tissue Expression Project (GTEx) database, the eQTL analysis results have shown that the homozygote majority of both rs2278528 and rs7594214 contributed to lower expression level of TTC32 in the coronary artery and the aorta artery (Figure 3A and 3B). Besides, the significantly higher TTC32 expression level of rs721932 CC subjects than that of GG subjects was observed in the tibial artery (Figure 3C). Similarly, research also reported that rs721932 was an eQTL of TTC32 in whole blood.^22^ Both artery and whole blood are responsible tissue of atherosclerosis. As stated above, TTC32 was indeed the new reasonable CAD susceptible gene.

### 3.6 False‐positive report probability results

To strengthen our results in the analysis for each genotype comparison for TTC32‐WDR35 gene cluster variants and CAD association, we performed the FPRP test and preset 0.2 as the FPRP threshold. All genotype comparisons with FPRP < 0.2 at a prior probability of 0.1 were considered noteworthy. The FPRP values for significant findings at different prior probability levels are shown in Table 5. For a prior probability of 0.1, the FPRP values were 0.080 for an association of rs721932 CG vs. CC genotype with a decreased risk of CAD in all individuals. The significant decrease of subgroup of male in CAD risk carrying rs721932 GG and CG genotypes remained noteworthy compared with CC genotype. Moreover, the association between rs721932 polymorphism (CG versus CC) and MI risk was also noteworthy, with a statistical power of 0.521 and the FPRP value of 0.017. By comparison, greater FPRP values were observed for the other significant associations, suggesting the lack of notable associations, which need further validation in larger studies.

**TABLE 5:** False‐positive report probability values for associations between the risk of CAD and the frequency of genotypes of TTC32‐WDR35 gene cluster in Chinese population

Genotype	Crude OR (95% CI)	*P* value	Statistical power	Prior probability				
				0.25	0.1	0.01	0.001	0.0001
rs12617744(T > A)								
AA vs.TT								
Male	0.62(0.42‐0.93)	.02	0.348	0.147	0.341	0.850	0.983	0.998
Smoker	0.45(0.23‐0.88)	.02	0.124	0.326	0.592	0.941	0.994	0.999
MI TA vs. TT	1.49(1.03‐2.15)	.04	0.545	0.180	0.398	0.879	0.987	0.999
CAD severity AA vs. AT vs. TT								
≥2VD vs. Con + 0VD	1.33 (1.02‐1.74)	.034	0.799	0.113	0.277	0.808	0.977	0.998
rs721932 (C > G)								
CG vs. CC	0.53 (0.36‐0.78)^d^	.001^d^	0.104^d^	0.028^d^	**0.080** ^d^	0.488^d^	0.906^d^	0.990^d^
MI CG vs.CC	0.68 (0.54‐0.86)^d^	.001^d^	0.521^d^	0.006^d^	**0.017** ^d^	0.160^d^	0.657^d^	0.951^d^
CG + GG vs. CC								
Male	0.68 (0.52‐0.89)	.004	0.515	0.023	**0.065**	0.435	0.886	0.987
>60 years old	0.79 (0.63‐0.99)	.04	0.923	0.115	0.281	0.811	0.977	0.998
Drinkers	0.53 (0.31‐0.89)	.02	0.208	0.224	0.463	0.905	0.990	0.999
CAD severity minor allele frequency								
≥2VD vs. Con + 0VD	078 (0.60‐1.00)	.048	0.881	0.140	0.329	0.844	0.982	0.998

Abbreviations: CI, confidence interval; MI, myocardial infarction; OR, odds ratio; VD, vessel disease.

^a^ Chi‐square test was used to calculate the genotype frequency distributions.

^b^ Statistical power was calculated using the number of observations in each subgroup and the corresponding ORs and *P* values in this table.

^c^ The level of false‐positive report probability threshold was set at 0.2 and noteworthy findings are presented.

^d^ Adjusted for age, sex, smoking, drinking, blood pressure and diabetes mellitus.

Bold values statistically significant probability of 0.1 under the preset prior probability <0.2.

## 4 DISCUSSION

The chromosomal region 2p24.1, harboring the TTC32‐WDR35 gene cluster, produced strong association signals in GWASs of CAD among different populations.^5^, ^8^ However, the complex patterns of LD structure in the locus yield obstacles to identifying the genuine underlying variants and corresponding genes accountable for these associations. In the current study, we not only reproduced the association of the region 2p24.1 with CAD in Chinese Han population but also uncovered several novel findings that were pertinent to this locus. Our results showed that the CG genotype of the SNP rs721932 was associated with overall risk of CAD. Based on unconditional logistic regression analysis with adjustment for conventional CAD risk factors, no statistically significant association with susceptibility to CAD was observed with other 4 SNPs. Stratification analysis additionally identified significant associations between rs12617744 genotype and CAD risk in male participants. Especially among males, the genotype AA of the SNP rs12617744 was strongly associated with a higher risk of CAD even after adjusting for conventional risk factors. A significant interaction was found between gender and rs12617744 in our study.

We also found that the increased gene dosage of rs721932 and rs12617744 minor alleles were significantly associated with CAD severity. Importantly, after adjusting for conventional risk factors, the CG genotype of rs721932 was significantly associated with MI risk, which is consistent with the association between the gene dosage of rs721932 and CAD progress. Moreover, both SNPs were significantly associated with HDL levels in the control group. It is widely acknowledged that HDL plays an important role in the development of CAD.^18^, ^22^ As stated above, the common variant rs7594214 and rs12617744 might contribute to the modulation of HDL levels and possibly to the severity of CAD through certain pathway in the Chinese Han population. Our data from FPRP analysis did strongly support the protective role of the G allele within the rs721932 polymorphism in the risk of CAD at a prior probability of 0.1. Nevertheless, we cannot obtain a relatively scientific conclusion regarding the potential links of rs12617744 and CAD risk.

The data for reported CAD‐associated SNPs, including rs16986953^8^ and rs2123536,^5^ were extracted from HapMap datasets for LD analysis. We found that the SNP rs12617744 was in moderate LD with rs16986953 and rs2123536 in Chinese Han population. Notably, the variant rs721932 displayed poor LD with these CAD‐associated SNPs with the *r* ^2^ < .01.

The SNP rs721932 lies in the first intron of AC079145.1 and about 1kb upstream of WDR35. The WDR35 gene, encoding a WD‐repeat protein, has been reported to be requisite for ciliary function, which plays a vital role in the transduction of signals in the hedgehog pathway.^23^, ^24^, ^25^, ^26^ Recent studies have shown that this pathway was unveiled to participate in cardiovascular phenotypes, such as myocardial ischemia.^27^ Also, a relevant study suggested that hedgehog signaling was also involved in the atherosclerotic process,^28^ which may partly explain the association between rs721932 and HDL regulation. As mentioned above, accumulating evidence has indicated that WDR35 is a feasible candidate gene involving in the polygenic model of CAD. Notably, AC079145.1 is overlapped with the promoter sequence of WDR35. Numerous studies have revealed that long non‐coding RNAs play important roles in various cellular functions by modulating expressions of targeted mRNAs at the transcriptional or post‐transcriptional levels.^29^ It has been widely reported that lncRNAs can regulate the expression of nearby protein‐coding genes.^15^ Thus, AC079145.1 might also participate in the expression regulation process of WDR35 and contribute to the mechanism of action underlying CAD. As stated above, considerable experimental evidence in vivo or vitro are urgently needed to corroborate these postulations.

To date, increasing expression quantitative trait locus (eQTL) mapping studies have been conducted to identify candidate genetic variants or genes that contribute to complicated phenotypes.^30^, ^31^ The eQTL result was obtained from data in the GTEx database, demonstrating significantly higher TTC32 expression of rs721932 CC subjects than GG subjects in the tibial artery. Additionally, a study also reported that rs721932 was an eQTL of TTC32 in whole blood.^32^ Relevant studies have shown that TRP‐containing proteins, including the TTC32 coding product, were involved in multiple regulatory processes by mediating other cochaperones.^9^ In silico analysis using UCSC Genome Browser annotations located a DNase I hypersensitivity site near to rs721932 in multiple cell lines. HaploReg data indicates that rs721932 is located in a region with enhancer or promoter histone marks among different cell lines. It indicated that the locus might have regulatory properties with the G allele of rs721932, leading to reduced expression of TTC32. As stated above, we might assume that rs721932 is more likely to affect the transcriptional activity of TTC32 gene and cis‐regulate its expression, contributing to the pathogenesis of CAD. Although SNPs rs2278528 and rs7594214 were demonstrated to cis‐regulate the expression level of TTC32 in the coronary artery, they were not associated with CAD risk in Han Chinese, probably due to ethnic specificity.

There are three limitations in our study that must be acknowledged. Firstly, all of the participants were recruited from one Chinese southwest city, and there were considerable differences in lifestyle and varied genetic frequency between Southern and Northern Han Chinese populations. Hence, the samples involved in our study may not be sufficiently representative of the general Han Chinese population. Secondly, the collection of family history information, an important factor for CAD, was limited, which might bias our results. Thirdly, this was a retrospective study, and thus, a possible selection bias might exist. Lastly, the controls included in the present study were not evaluated by coronary angiography, some of whom may have unperceivable CAD. Therefore, further large prospective cohort studies with the more rigorous experimental design are demanded to corroborate our results.

## 5 CONCLUSIONS

The results of the present study demonstrated that the novel variants rs721932 and rs12617744 at the TTC32‐WDR35 gene cluster are significantly associated with predisposition to CAD in Chinese Han population, and both the SNPs might contribute to modulation of HDL levels and possibly to the severity of CAD. Our results also provide evidence for WDR35 or TTC32 as a strong candidate gene underlying the pathogenesis of CAD. Further studies are necessary to validate our findings and to unveil the potential biological mechanism.

## CONFLICT OF INTEREST

The authors declare no relationships that could be construed as a conflict of interest.

## AUTHOR CONTRIBUTIONS

YX, YZ, and M‐LY searched the literature, selected the study, analyzed data, and drafted the article. M‐ML carried out the epidemiological survey, collected the samples, and helped to draft the manuscript. X‐JT took part in assessments of quality. LZ designed the study, interpreted data, and revised the article. All authors read and approved the final manuscript.

## ETHICS APPROVAL AND CONSENT TO PARTICIPATE

The Ethics Committee of Chongqing Medical University approved the present study, and written informed consent was received from each subject.

## Supporting information

Supplementary Material

## Data Availability

The data used to support the findings of this study have not been made available because the data also forms part of an ongoing study.
