# Supplementary material for: Association study of genetic variants at TTC32‐WDR35 gene cluster with coronary artery disease in Chinese Han population
Source: J Clin Lab Anal. 2020 Oct 2;35(2):e23594. doi: 10.1002/jcla.23594 (PMC7891520; doi:10.1002/jcla.23594)
Supplement: Supplementary file 1 — Supplementary Material [file JCLA-35-e23594-s001.docx]

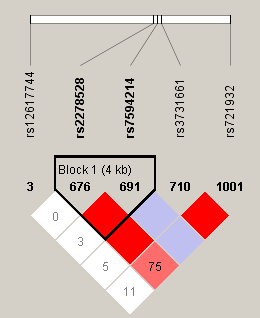


Supplymentary Figure 1 Linkage disequilibrium (D') for identified 5 SNPs on the TTC32-WDR35 gene Cluster, as generated by Haploview 4.2 from the genotype data on 103 samples of Chinese Han descendant. The dark colors indicate higher levels of LD, while the light color indicates lower LD. The “r” value is indicated inside the diamonds.

| Supplementary Table 2. Distribution of the Five Analyzed SNPs in the TTC32-WDR35 Gene Cluster. | | | | |
| --- | --- | --- | --- | --- |
| SNPs | Gene | Chr. Pos. | Gene Pos. | MAF (HapMap-CHB) |
| rs12617744 | TTC32 | 19757672 | 3’ flanking | 0.442 |
| rs2278528 | AC013400.1 | 19902071 | exon | 0.374 |
| rs7594214 | WDR35 | 19906608 | 3’ flanking | 0.146 |
| rs3731661 | WDR35 | 19911095 | intron | 0.204 |
| rs721932 | AC079145.1 | 19991137 | intron | 0.252 |

| Supplementary table 3. Associations of SNPs rs12617744 and rs721932 with risk of MI in a Chinese Han population. | | | | | |  |
| --- | --- | --- | --- | --- | --- | --- |
| Genotype | MI | Control | OR(95%CI) | P | adjusted OR* (95%CI) | P* |
| rs12617744(T>A) |  |  |  |  |  |  |
| TT | 51(27.4) | 314(35.7) | 1 |  | 1 |  |
| TA | 100(53.8) | 414(47.1) | 1.49(1.03-2.15) | 0.04 | 1.43(0.94-2.19) | 0.10 |
| AA | 31(16.7) | 151(17.2) | 1.27(0.78-2.07) | 0.34 | 1.57(0.90-2.73) | 0.11 |
| rs721932(C>G) |  |  |  |  |  |  |
| CC | 116(63.0) | 486(52.9) | 1 |  | 1 |  |
| CG | 59(32.1) | 393(42.8) | 0.63(0.45-0.88) | 0.01 | 0.53(0.36-0.78) | 0.001 |
| GG | 9(4.9) | 39(4.2) | 1.01(0.48-2.15) | 0.98 | 1.04(0.44-2.46) | 0.93 |

* ORs and P values were calculated by logistic regression analysis with adjustment for age, sex, smoking, drinking, blood pressure and diabetes mellitus.

| Supplementary table 4.Stratification and interaction analysis between SNP rs12617744 genotypes and risk of CAD. | | | | | |
| --- | --- | --- | --- | --- | --- |
| gender | rs12617744(T>A) | | | | P interaction |
|  | Cases N (%) | Controls N (%) | Adjusted OR* (95% CI) | Adjusted P* |  |
| male |  |  |  |  | 0.01 |
| TT | 163(37.6) | 152(34.4) | 1 |  |  |
| TA | 214(48.3) | 206(65.6) | 0.95(0.67-1.36) | 0.79 |  |
| AA | 56(12.6) | 84(19.0) | 0.59(0.36-0.97) | 0.04 |  |
| female |  |  |  |  |  |
| TT | 153(33.5) | 158(36.9) | 1 |  |  |
| TA | 223(48.8) | 205(47.9) | 0.97(0.69-1.38) | 0.88 |  |
| AA | 81(17.7) | 65(15.2) | 1.60(0.98-2.63) | 0.06 |  |
| *ORs and P values were obtained from a logistic regression model with adjustment for age, gender, smoking, drinking, blood pressure and diabetes mellitus. | | | | | |
